# Unexpected Result in a Case of Strangulated Hernia

**Published:** 1884-03

**Authors:** Geo. R. West

**Affiliations:** Rome, Ga.


### UNEXPECTED RESULT IN A CASE OF STRANGULATED HERNIA.

BY GEO. R. WEST, M.D., ROME, GA.

I was called to see A. H., a colored boy, aged 23 years, on the night of December 28th, 1883. I went immediately and found him suffering very much with a strangulated, direct inguinal hernia.

The boy drove a coal delivery wagon, and was much subjected to strains in loading and unloading. He had not been working the day previous to my visit, but had walked to the country, about two miles, and during the afternoon, about two o'clock, while straining at stool, he felt the bowel escape. He managed to walk home, however, and went to bed, hoping time would cure him, but the pain and size of tumor increasing, he sent for me at eleven o'clock. Before I arrived he had vomited twice with "purge," as his old attendant called it, in the vomited matter His pulse was sixty per minute; he was sweating, though the night was cold, and his general symptoms did not indicate immediate danger. I vainly attempted to replace the bowel, both by position and taxis, persisting in my taxis only about seven or eight minutes. I then gave him the third of a grain of morphine, hypodermically, and left him for an hour. At the expiration of the hour, I returned with chloroform and an assistant, and again attempted taxis with no better success than before. I then thoroughly chloroformed him and applied taxis vigorously, with no better result. I left, with directions to his only attendant to apply hot clothes over the tumor, to repeat them frequently, and to give him twenty drops of laudanum every three hours.

When I saw him next morning I was vexed to find my directions had not been carried out, on account of the attendant preferring to sleep.

Seriously contemplating an operation, I called a consultation. We tried taxis again, and failing, decided against an immediate operation, but to continue the Tr. Opii. and warm cloths. This treatment commenced on the morning of the 29th December, 1883, and was persisted in until January 1st, 1884, when I gave up in disgust and determined to operate.

Some may wonder why I persisted so long, but the reason was that at each visit—and I made about three a day during this period—

he seemed doing so well, and the tumor growing more soft and somewhat diminished in size. Tuesday morning, January 1st, 1884, I operated, using ether. I cut down upon the sac, and, with a probe-pointed bistoury, passed along my finger to the point of constriction, cut the conjoined tendon until everything seemed loose. I then attempted to put back the extended viscus, but was surprised to find it immovable. The adhesions around the bowel, after opening the sac of the hernia, were found to be so strong and numerous, and extending so far down into the scrotum, that to tear them loose seemed certain death to my patient, and I did not have the courage to continue. All the various surgical operations justifiable under the circumstances occurred to me, but I being young and inexperienced, preferred to close up my incision and let my patient die, as I certainly expected he would. I kept him after the operation for many days under the influence of opium and on a liquid diet. His condition never became alarming, pulse never higher than 110, and temperature never more than 101°. On the 9th of January he took some laxative pills, and on Thursday had a very complete evacuation of the bowels, with no pain or blood. The bowels then became regular, one action a day, and have so continued. Pus had been flowing from my incision for a week, but still the tumor remained about the same size. On Saturday morning, a pimple was noticed at the most dependent portion of the scrotum, which burst during the day and ran freely. At my visit in the evening, I enlarged the opening and drew forth a piece of bowel half the circumference of the gut and about two inches long. The veins in this piece of slough were enlarged and tortuous. The next day I took some omentum from the same place, and gradually the whole tumor, composed of intestine and omentum, sloughed off and was discharged at this orifice. The patient has fully recovered.

At the time of the hernia, he was under my treatment for chancroid, which had been touched with nitric acid, but which received no more attention after I began the treatment of the hernia. I am glad to state it has healed nicely of its own accord.

